# An Analysis of the Neurological and Molecular Alterations Underlying the Pathogenesis of Alzheimer’s Disease

**DOI:** 10.3390/cells10030546

**Published:** 2021-03-04

**Authors:** Chantal Vidal, Li Zhang

**Affiliations:** Department of Biological Sciences, University of Texas at Dallas, Campbell Road, Mail Stop RL11, 800 W, Richardson, TX 75080, USA; cxv130930@utdallas.edu

**Keywords:** Alzheimer’s disease, mitochondria, heme, amyloid beta

## Abstract

Alzheimer’s disease (AD) is a neurodegenerative disorder characterized by amyloid beta (Aβ) plaques, neurofibrillary tangles, and neuronal loss. Unfortunately, despite decades of studies being performed on these histological alterations, there is no effective treatment or cure for AD. Identifying the molecular characteristics of the disease is imperative to understanding the pathogenesis of AD. Furthermore, uncovering the key causative alterations of AD can be valuable in developing models for AD treatment. Several alterations have been implicated in driving this disease, including blood–brain barrier dysfunction, hypoxia, mitochondrial dysfunction, oxidative stress, glucose hypometabolism, and altered heme homeostasis. Although these alterations have all been associated with the progression of AD, the root cause of AD has not been identified. Intriguingly, recent studies have pinpointed dysfunctional heme metabolism as a culprit of the development of AD. Heme has been shown to be central in neuronal function, mitochondrial respiration, and oxidative stress. Therefore, dysregulation of heme homeostasis may play a pivotal role in the manifestation of AD and its various alterations. This review will discuss the most common neurological and molecular alterations associated with AD and point out the critical role heme plays in the development of this disease.

## 1. Introduction

Dementia is a chronic dysfunction of cortical and subcortical function that causes cognitive decline [1]. It affects about 5% of the elderly population over the age of 65 [1]. Alzheimer’s disease (AD) is the most common and most studied cause of dementia [2]. In Europe and North America, AD is more common than vascular dementia [1,3,4]. One study in Shanghai noted that 65% of all dementias were clinically diagnosed as AD [5]. AD is a progressive neurodegenerative disorder that affects memory and other cognitive functions. It is the 6^th^ leading cause of death in the United States, and more than 5 million Americans are currently living with this disease [6]. In the US, the number of people with this disease is projected to double by 2050 [6]. Worldwide, 50 million people are living with this disease, and by 2050, this community is likely to rise to about 152 million people [7]. Therefore, developing an effective treatment or cure for this disease is essential.

AD can be divided into two subgroups: late and early onset forms of this disease [8]. Early-onset AD, also known as familial AD (FAD), affects individuals under 65 years of age and only accounts for 2–10% of the total cases of AD [9]. This form of AD is attributed to mutations in genes such as the amyloid precursor protein (*APP*), Presenilin 1(*PSEN1*), and Presenilin 2 (*PSEN2*) [10,11,12,13,14,15,16]. Late-onset AD is considered sporadic (SAD), although genetic risk factors have been identified, including the apolipoprotein E gene (*APOE*) [17]. Regardless of the type of AD, there are specific pathologies that are attributed to this disease, which include the presence of extracellular plaques made of insoluble amyloid beta peptides (Aβ) and neurofibrillary tangles (NFT) [6,10,18,19]. Recently, mitochondrial dysfunction, reduced energy metabolism, synaptic loss, altered Wnt signaling, and inflammation have been implicated in AD [20,21,22].

The US Food and Drug Administration (FDA) has approved only a few drugs to treat AD, and they include memantine, donepezil, galantamine, and rivastigmine [23,24,25,26]. These drugs either regulate glutamate activity, a chemical involved in information processing, or delay the breakdown of acetylcholine, a chemical in the brain essential for memory. Unfortunately, these drugs only moderately delay cognitive symptoms, and approximately half of the people who take these drugs do not respond to them [26,27]. To develop effective therapies that can slow down the cognitive symptoms of AD and halt the disease’s overall progression, we must understand the molecular alterations that initiate the cascade of events leading to neuronal dysfunction in AD.

One specific alteration that can play a pivotal role in the development of AD is dysfunctional heme homeostasis. Heme is likely a common factor that links several metabolic alterations in AD, including dysregulated iron metabolism, decreased mitochondrial complex IV levels, and increased levels of oxidative stress [28,29,30]. Heme deficiency induced in two human brain cell lines caused reduced mitochondrial complex IV expression, altered APP expression, and corrupted iron homeostasis [31]. Furthermore, in a 2019 study, the presence of anemia was associated with a 41% increased risk for AD [32]. Therefore, this review will discuss the various alterations seen in AD and point out the critical role heme plays in AD pathogenesis.

## 2. Genetic Risk Factors

Family history has shown to increase a person’s chance of developing AD. There are various genetic risk factors associated with the development of AD. These risk factors are usually associated with some of the histological alterations previously discussed. The presence of *APP, PSEN1*, and *PSEN2* mutations as well as other genetic risk factors have been attributed to an increased risk of developing AD.

### 2.1. APP, PSEN1, and PSEN2 Mutations

In 1984 Dr. Glenner and Dr. Wong isolated and identified APP, but it was not until 1991 and 1996 that mutations in the *APP, PSEN1*, and *PSEN2* genes were identified as having a causative role in the production of Aβ peptides and senile plaques [33,34,35]. Mutations in these genes are commonly associated with Familial Alzheimer’s disease (FAD).

**Figure 1:**
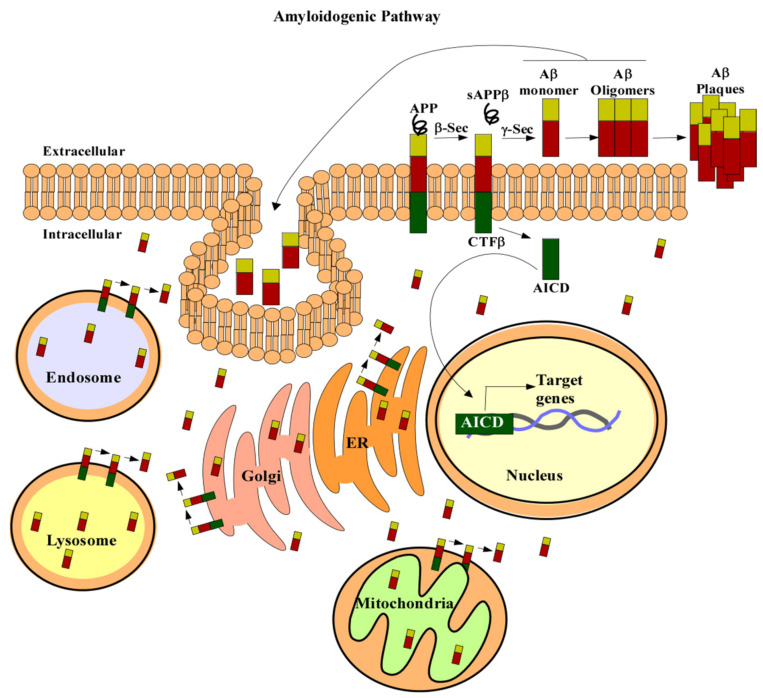
APP processing. In the amyloidogenic pathway APP (in green red and gold) is cleaved by β secretase to produce a soluble form of APP, sAPPβ. Then, γ-secretase cleaves the remaining amino acid protein CTFβ to produce Aβ (red and gold) and AICD (green). Aβ can then form oligomers and plaques which are characteristic of AD. AICD can translocate to the nucleus (beige) and regulate gene expression (DNA is in grey and blue). APP can also be localized to the trans-Golgi network (pink), ER (orange), endosomal (blue), lysosomal (yellow), and mitochondrial membranes (green). Aβ liberation can occur wherever APP and the β- and γ-secretases are localized. Aβ can also be taken up by the cell to form intracellular pools. Abbreviations: amyloid precursor protein (APP), secreted APP derivative (sAPPβ), amyloid beta (Aβ), β-Secretase-Derived C-Terminal Fragment (CTFβ), APP intracellular domain (AICD), β-APP-site cleaving enzyme (β-Sec), γ-Secretase (y-Sec), ER (endoplasmic reticulum).

In the amyloidogenic pathway, APP is cleaved by β- and γ-secretases to produce a 4kDa protein, known as Aβ (Figure 1) [33,36,37,38]. The first proteolytic cleavage is produced by the membrane-bound aspartyl protease, β-APP-site cleaving enzyme (BACE). This protease renders a secreted APP derivative, sAPP, and a membrane-bound protein fragment of 99 amino acids, β-Secretase-Derived C-Terminal Fragment (CTFβ) [38,39,40,41]. CTFβ is further cleaved by γ-secretase containing the four proteins: APH1, PEN2, nicastrin, and presenilin (PS1 or PS2). Cleavage of CTFβ produces different lengths of Aβ peptides and an APP intracellular domain (AICD) [36,38]. The 40-residue peptide, Aβ40, makes up the majority of the total Aβ produced in cells [38]. Less than 5% of the generated Aβ ends at the residue 42 [38]. Aβ42 has a higher rate of fibrilization and insolubility and therefore is more prevalent in senile plaques [42,43]. The increased ratio of Aβ42/Aβ40 is one of the pathogenic hallmarks of AD [42,44,45,46,47,48].

*APP* gene mutations usually involve those in the β-APP-site cleaving enzyme (BACE) cleavage site, those at the γ-secretase cleavage site, and those in the mid domain Aβ region [49]. Mutations in this gene can render either an increase in Aβ42 produced or an increase in Aβ42/Aβ40 ratios [50,51]. Furthermore, in 2016 a detailed study carried out by Sun et al. [52] characterized 138 distinct *PSEN1* mutations and their effect in Aβ production. This study revealed that 34 variants increased production of AB42 while the other 104 caused a reduction in the total production of Aβ40 and Aβ42 [52]. More importantly, the production of Aβ40 was typically more affected than the production of Aβ42, leading to the elevated ratios of Aβ42/Aβ40 [52,53,54]. Similarly, mutations in *PSEN2* increase Aβ42/Aβ40 ratios, suggesting that the shift in the Aβ ratios has a role in developing FAD [55]. An early study analyzing SAD and FAD brains found that the ratio of the long-tail form of Aβ to total Aβ was increased in FAD brains [56]. Aβ42/Aβ40 ratios were also elevated in FAD mutant induced pluripotent stem cells (IPSC) relative to controls [57]. APPsw mice expressing apoE4 also exhibited increased Aβ42/Aβ40 ratios [58]. Furthermore, Aβ can be generated outside the central nervous system (CNS), contributing to the circulating Aβ pool [59]. Roher et al. [59] found that brains and skeletal muscles from AD patients express significantly more Aβ than non-demented controls.

### 2.2. ApoE4

**Figure 2:**
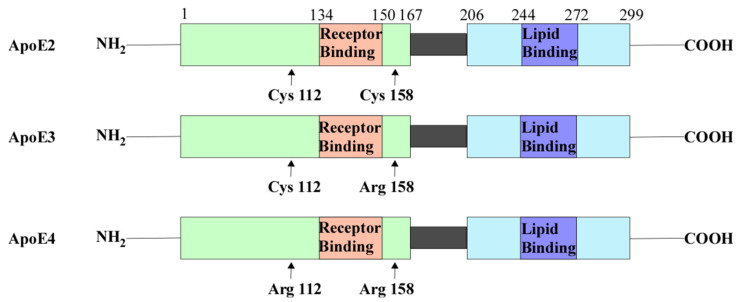
ApoE contains the N-terminal domain which contains the receptor-binding region and the C-terminal domain containing the lipid-binding region. There are three isoforms of APOE: ApoE2, ApoE3, and ApoE4. ApoE2 contains Cys residues at positions 112 and 158. ApoE3 contains a Cys residue at position 112 and an Arg residue at 158. ApoE4 contains Arg residues at both of these positions. Abbreviations: cysteine (Cys), arginine (Arg), apolipoprotein E(ApoE).

ApoE is a glycoprotein known to regulate the clearance of lipoproteins from the plasma by serving as the ligand that binds to various cell surface receptors [60]. These receptors then internalize apoE-containing lipoprotein particles. ApoE has three isoforms (Figure 2): apoE2, apoE3, and apoE4. Amino acid sequencing of these isoforms showed that they differ in the residues at positions 112 and 158 (Figure 2) [61,62]. These variations in amino acid residues affect their receptor and lipid-binding affinities. For example, apoE2 has Cys residues at both positions and has a preference for high-density lipoproteins (HDL) [61,62]. ApoE2 also has a low binding affinity to low-density lipoprotein (LDL) receptors compared to apoE3 [62,63]. ApoE3 has a Cys residue at position 112 and an Arg residue at 158. Similar to ApoE2, APOE3 can preferentially bind to HDL [62]. *APOE4* has Arg residues at both positions that allow a higher binding affinity to LDL receptors and larger triglyceride-enriched lipoproteins (TRL) [62,64]. The differences in binding affinities affect their role in lipid metabolism. For example, apoE3 is associated with cholesterol efflux and the formation of APOE-containing HDL, while apoE4 accumulates in the endosomal compartments causing impaired cholesterol efflux, leading to the generation of Aβ [65,66,67,68,69].

ApoE2 can have a protective role against the development of AD [70]. Macrophages expressing apoE2 are more efficient in degrading Aβ than those expressing apoE3 or apoE4 [71]. A meta-analysis carried out in 2015 showed that carriers of the *APOE2* allele have a lower rate of brain amyloid presence than *APOE3* carriers [72]. ApoE3 is the most common form of apoE and plays a neutral role in AD.

ApoE4 exists in approximately 20% of the population and is the most significant genetic risk factor for SAD [62,70]. The association of apoE4 with late-onset Alzheimer’s disease was first discovered in three landmarks studies published in 1993 [73,74,75]. One of these studies showed that apoE has a high affinity to Aβ and that the *APOE4* allele has a higher association with AD [74]. Corder et al. [73] found that the risk for AD increases from 20% to 90% with increasing number of *APOE4* alleles in 42 families with late-onset AD. Furthermore, the *APOE4* allele was shown to decrease the mean age of onset from 84 to 68 years [73].

Studies done on human induced pluripotent stem cells showed that *APOE* upregulates APP expression, and this expression is most prominent for *APOE4*, followed by *APOE3*, and finally *APOE2* [76,77]. The *APOE4* allele is also associated with increased amyloid deposition and NFTs. [78,79]. Mitochondrial dysfunction has also been studied in *APOE4* carriers. In a study analyzing the neurotoxicity of apoE4 fragments on cultured Neuro-2a cells, apoE4 fragments formed filamentous inclusions in some cells that interacted with mitochondria causing mitochondrial dysfunction [80].

### 2.3. Other Genetic Risk Factors of AD

A Genome-wide association study (GWAS) of 74,046 individuals identified 11 genes associated with AD [81]. These genes include *APOE*, *TREM2*, *CD33*, *BIN1*, *CLU*, *CR1*, *MS4*, *CD2AP*, *ABCA7*, *PICALM*, and *EPHA1* [81]. Among these genes, *TREM2* variants cause a two-fold increase in the risk for AD [82,83]. TREM2 is primarily expressed in microglia and helps mediate phagocytosis, inhibit inflammatory signals, and promote cell survival [82]. TREM2 activation can lead to ligand binding, inducing a signal cascade that results in increased phagocytosis and decreased pro-inflammation [82]. Therefore, a compromised function of TREM2 may lead to decreased clearance of cell debris and possibly the removal of Aβ in Alzheimer’s disease [83]. In 2019, Parhizkar et al. [84] found that in the absence of TREM2, amyloid plaque seeding increased and microglia clustering around newly seeded plaques decreased.

CD33 is significantly upregulated in AD patients’ brains and can modulate microglial activation and inhibit Aβ clearance [85]. *BIN1* has also been linked to AD in early GWAS and is the most important genetic susceptibility locus in AD after *APOE* [86]. An analysis of 114 AD brain tissues and 167 control brain tissues showed an increased expression of BIN1 in AD brains [87]. Furthermore, loss of the Drosophila *BIN1* ortholog *AMPH* was able to suppress Tau-induced neurotoxicity [87]. This suggests that *BIN1* acts as a genetic risk factor for AD by regulating Tau pathology.

*CLU* and *CR1* genes, previously identified as having a role in Aβ clearance, were associated with the development of AD in a GWAS of 2032 AD patients and 5328 controls [88,89,90]. MS4 family proteins have also been implicated in the pathogenesis of AD [91]. Karch et al. [92] showed that *MS4A6A* expression correlates with neuropathological measures of AD. Similarly, single nucleotide polymorphisms (SNPs) in the *CD2AP* gene are associated with the development of SAD [93,94]. The CD2AP protein can modulate Tau-mediated neurotoxicity, regulate Aβ generation, and maintain the blood–brain barrier integrity [93]. For example, Cochran et al. [95] analyzed CD2AP deficient mice and found that these mice exhibit reduced blood–brain barrier integrity, suggesting a cardiovascular role in AD. Loss of function variants in the *ABCA7* gene, involved in the Aβ clearance pathway, have also been implicated in the development of AD [94,96,97]. Sakae et al. [98] found that ABCA7 deficiency alters the brain lipid profile and impairs memory. Furthermore, APPPS1 deficient for ABCA7 had an increased amyloid plaque burden.

Several GWAS have identified variants within the *PICALM* gene as risk factors for developing AD [94,99,100]. PICALM, involved in clathrin-mediated endocytosis, likely plays a role in APP endocytosis and thus regulates Aβ generation [101]. A 2011 study analyzing four GWA datasets found *EPHA1* variants implicated in AD [94]. Having a minor C allele at SNP (rs11771145) on the *EPHA1* gene is associated with a lower chance of being Aβ positive, suggesting its protective role in preventing AD [102]. A meta-analysis of 30,000 subjects and a large GWAS associated multiple variants in the *SORL1* gene with both late- and early-onset AD [103,104]. Furthermore, *SORL1* mutations have been associated with a weakened interaction between the SORL1 protein and the full-length APP, altering the levels of APP trafficking [105]. Overall these newly identified genetic risk factors suggest new genetic and molecular mechanisms underlying AD’s pathogenesis.

## 3. Neurological and Molecular Alterations of AD

AD is characterized by various histological and molecular alterations. The most established hallmarks of this disease include amyloid plaques and neurofibrillary tangles [106]. Despite the extensive interest in amyloid plaques and neurofibrillary tangles, several other alterations are associated with this disease [17,28,107,108,109,110,111,112]. Unfortunately, there is much debate on which of these alterations play a causative role in the development of AD. This section will focus on the neurological and molecular alterations implicated in AD.

### 3.1. Amyloid Beta

The pioneering work of Dr. Alios Alzheimer started in 1906 when a patient of the Community Psychiatric Hospital at Frankfurt named Auguste D. died [113]. The patient presented various cognitive impairments, including memory loss and confusion [113]. Dr. Alzheimer analyzed the brain of Auguste D. and discovered the histological alterations that would later be known as plaques and neurofibrillary tangles (NFT) [113,114]. These senile plaques are made of the accumulation of a 39–42 amino acid peptide called amyloid beta (Aβ) [46,115,116]. The characteristic accumulation of this Aβ protein in AD patients has caused many researchers to believe that this histological alteration is the cause of the disease.

Aβ is well-known to play a pivotal role in AD pathology, but the exact mechanism has been widely debated. Nuclear magnetic resonance has shown that Aβ42 can form oligomers that incorporate into the cell membrane and form channels that are highly permeable to Ca^2+^ [117,118]. This causes a disruption in calcium homeostasis, which induces synaptic degeneration [118,119]. Aβ can also cause neuronal death in vivo through the caspase 3 apoptotic cascade [120]. One study utilizing CK-p25 mice expressing increased Aβ levels showed differentially expressed genes enriched in cell cycle, immune response, and synaptic functions compared to controls [21]. It has also been proposed that Aβ42 causes neuronal apoptosis by activating the caspase pathway, thereby promoting mitochondrial fission and increasing reactive oxygen species (ROS) [121].

Despite ample evidence of the toxicity of Aβ, there is a poor correlation between the clinical symptoms in sporadic AD and Aβ plaque deposition [122]. This has caused critics to suggest that Aβ does not mediate AD [122]. However, in FAD cases, in which disease pathogenesis is more clearly driven by Aβ, the anatomical correlation between plaques and neuronal loss is consistent with that of SAD. This implies that Aβ can still drive neuronal loss without the colocalization of plaques and neurodegeneration [122,123]. Furthermore, the poor correlation between fibrillar Aβ and neuronal loss in SAD can be attributed to the different aggregation states of Aβ. There is some evidence that Aβ oligomers correlate well with AD severity [124]. Aβ oligomers can cause neuron degeneration and hyperphosphorylation of tau, key characteristics of AD [125].

Accumulation of intracellular Aβ is also associated with AD. Two mechanisms have been proposed for accumulating intracellular Aβ (Figure 1): intracellular sites of Aβ production or reuptake of Aβ [126]. APP can be localized to the trans-Golgi network, ER, endosomal, lysosomal, and mitochondrial membranes [126]. Aβ liberation can occur wherever APP and the β- and γ-secretases are localized. Therefore, if APP cleavage occurs within the cell, Aβ can accumulate intracellularly. Extracellular Aβ can also be taken up by cells to form intracellular Aβ pools [126,127]. In SH-SY5Y, the uptake of Aβ40 and Aβ42 occurs exclusively via endocytosis [127]. There are also several putative receptors and transporters associated with the accumulation of intracellular Aβ [128,129,130,131]. For example, binding of Aβ to the scavenger receptor for advanced glycation end products (RAGE) can cause internalization of Aβ [132]. Similarly, the G protein-coupled formyl peptide receptor-like 1 (FPRL1) and the NMDA receptors can also uptake Aβ [133,134].

Studies utilizing the Tg2576 mouse model have shown that accumulation of intracellular Aβ can lead to synaptic dystrophy [135]. Intracellular Aβ is also associated with decreased mitochondrial membrane potential [136]. Furthermore, injections of Aβ in primary neurons have also been shown to cause significant cell death through the p53-Bax cell death pathway [137]. These studies suggest that the accumulation of intracellular and extracellular Aβ and the different physical and aggregated states of Aβ play a role in neuronal damage, leading to the development of AD.

### 3.2. Neurofibrillary Tangles

Neurofibrillary tangles (NFTs) are filamentous aggregates of the microtubule-associated protein tau [138]. Tau is involved in microtubule stability and cytoskeletal trafficking within mature neurons [139]. Tau has also been seen to copurify with tubulin and plays a major role in polymerization and hence microtubule assembly [140]. Tau is tightly regulated by various post-translational modifications, but phosphorylation is the most noted. In the brain, tau is predominantly expressed in neurons, and its non-phosphorylated form is restricted to axons [141]. Previous studies on tau revealed that specific modes of phosphorylation can cause conformational changes that affect its ability to polymerize tubulin [142]. Phosphorylation at Thr231, Thr214, and Ser235 causes dissociation of tau from microtubules [143,144]. Interestingly, phosphorylation of tau at the C-terminal region causes self-aggregation [145]. This role of phosphorylated tau can contribute to the formation of NFTs.

One of the histological characteristics of AD is the presence of NFTs composed of hyperphosphorylated tau [146,147,148]. NFTs have been shown to correlate well with disease progression [149]. It has also been proposed that NFTs can directly cause damage to neurons and glial cells by displacing cytoplasmic organelles to the periphery, inhibiting proteasome activity, or disturbing microtubule assembly [147,150,151]. Furthermore, oligomeric tau has been shown to induce neurodegeneration by decreasing levels of mitochondrial respiratory complex I activity [152]. NFTs can also prevent mitochondrial transport, causing oxidative stress and energy deprivation, which in turn leads to neurodegeneration [153,154]. The role of NFTs and their function in either accelerating or halting neurodegeneration has also been widely debated [155,156]. A study by Ferrari et al. [157] found that tau was likely to be aggregated in cells treated with Aβ, suggesting that tau pathology follows Aβ toxicity in AD.

NFTs are also known to induce oxidative stress [158]. Mitochondria are the main source of oxidative stress, and the mitochondrial superoxide dismutase 2 (sod2) plays a critical role in alleviating ROS. To determine if oxidative stress causes NFTs, a study utilizing sod2 null mice showed that increasing amounts of antioxidants significantly reduced levels of hyperphosphorylated tau [159]. This suggests that mitochondrial oxidative stress plays a role in the histological alteration of tau [159]. A Quantitative analysis also showed that neurons with NFTs have a 40–56% decrease in the levels of 8-hydroxyguanosine (8OHG), suggesting that NFTs help reduce levels of oxidative stress in neurons [158,160].

### 3.3. Neuronal Loss/Synaptic Loss

Neuronal loss is a prominent pathological feature of AD. AD is considered a neurodegenerative disease which means the clinical manifestation of AD is correlated with neuronal loss [111]. There are various mechanisms that might contribute to the loss of neurons seen in AD. For example, studies have shown that Aβ is attributed to the progression of AD because of its cytotoxicity [161]. Similarly, mitochondrial dysfunction and oxidative stress might also play a critical role in neuronal death [107]. Despite the debate on the mechanism, neuronal death is a key characteristic of AD.

Electron microscopy has also demonstrated a correlation between synapse counts and scores on the Mini-Mental State Examination. Increased synaptic loss is linked to lower mental status scores [162,163]. The synaptic markers synaptophysin and syntaxin and postsynaptic density-95 are known to decrease with age in 5xFAD mice [11]. A meta-analysis of 57 synaptic markers revealed a consistent synaptic loss across the hippocampus and frontal cortex. Specifically, the presynaptic markers were seen to be more affected [164].

### 3.4. Blood–Brain Barrier Dysfunction

The blood–brain barrier (BBB) refers to the microvasculature of the central nervous system. The BBB serves to separate the CNS from the peripheral tissue. Specifically, the BBB is known to regulate the neural microenvironment by mediating the entry and exit of various substances, including metabolites, toxins, and inflammatory mediators [165]. The endothelial cells that make up the blood vessels of the CNS have tight junctions that limit vesicle-mediated transcellular transport and transporters [166]. There are two categories of transporters in CNS endothelial cells, and they include efflux transporters that transport lipophilic molecules to the blood and nutrient specific transporters that allow uptake of nutrients to the CNS. The nutrient-specific transporters also help remove waste from the CNS [167]. Furthermore, these endothelial cells contain high mitochondrial levels that can drive the ion gradient necessary for transport functions [168]. Another important concept of the BBB is the presence of collagen, laminin, nidogen, heparin, and other secreted molecules that provide an additional barrier [167]. Regardless of the tight regulation of the BBB, various studies have shown that BBB dysfunction is correlated to AD progression.

One such hypothesis of the neurovascular dysfunction in AD is that increased Aβ in the brain interstitial fluid (ISF) is due to decreased Aβ clearance or increased levels of Aβ influx receptors [169]. Studies done on the Aβ clearance receptor, lipoprotein receptor-related protein (LRP), show that Aβ causes proteasome-dependent degradation of LRP, resulting in the low levels of LRP seen in AD patients. In WT mice, the Aβ influx receptor, RAGE, decreases cerebral blood flow (CBF) with the addition of Aβ [130]. Moreover, cerebral blood flow is decreased in some areas of the brain over 50%, leading to reduced Na/K pup activity and glutamate release [170,171,172]. Other studies propose that decreased cerebral blood flow is caused by a decrease in blood vessel diameter, particularly around senile plaques [173]. A study analyzing vascular smooth muscle cells (VSMC) in AD revealed that the hypercontractile phenotype of VSMCs could lead to the hypoperfusion seen in AD [174]. Other studies have shown a breakdown of the BBB with leakage of blood-borne molecules [169]. All these characteristics have been proposed to induce or contribute to the cognitive decline seen in AD.

### 3.5. Inflammation

Neuroinflammation is defined as an immune system response characterized by the activation of glial cells and the production of inflammatory mediators. [175,176]. Molecular networks constructed from whole-genome gene-expression data of 1647 postmortem brain tissues from late-onset AD patients revealed a strong association between activation of the immune system and AD pathology [177]. Inflammatory cytokines have also been reported to increase in disease progression or during the conversion of mild cognitive impairment (MCI) to AD [178,179]. A microarray study of young, aged, and AD cases showed an upregulation of innate immune system pathways in aging brains and a modest upregulation of these genes in AD [178]. These results suggest that inflammation is likely an early event in the preclinical stages of AD [178].

Among the innate immune cells, microglia play an important part in neuroinflammation [180]. Immunostaining of microglia in postmortem brain sections found an increase in microglia detection in mid-to-late stage AD [181]. Aβ can bind to various receptors expressed in microglia and can result in the production of inflammatory cytokines and chemokines [182]. Activated microglia can also cause neurotoxicity by releasing superoxide free radicals, NO, and TNFα [183,184]. Furthermore, microglia are known to play a critical role in the removal of Aβ [182]. However, studies suggest that microglia can lose their Aβ-clearing capabilities in AD [185,186].

Aβ and tau-containing NFTs can directly activate the classical complement pathway [109]. The classical complement system consists of a number of proteins and proteases that are activated in a cascade. Studies in AD patient brains have revealed an increase in immunoreactivity of C1q, C3b, C4d, C5b-9, and MAC surrounding senile plaques [187,188]. RNA sequencing and histological characterization of brain tissues revealed an upregulation of C3 in synapses of human AD brains with tau pathology [189]. Deletion of C3 was shown to rescue plaque-associated synapse loss in PS2APP mice and ameliorate neuronal loss [189]. These results show that the complement system contributes to neurodegeneration, and blocking C3 might be protective in AD.

### 3.6. Defective Cholesterol Metabolism

The brain has the highest cholesterol content of all organs, and it plays a critical role in the development and function of neurons [190]. The supply of neuronal cholesterol in adult brains is mainly produced by glial cells [190,191]. Apart from the biosynthesis of cholesterol, astrocytes can generate ApoE to combine with cholesterol and be secreted out of the cell by the activity of ATP-binding cassette transporters [192,193]. Neurons can then take up this complex utilizing LDL receptors, and the cholesterol can be stored to meet the need for neurons [193].

Defects in brain cholesterol have been implicated in AD [194]. One study showed that plasma cholesterol is 10% higher in AD patients compared to control subjects [195]. Epidemiological evidence has also confirmed that elevated cholesterol is a risk factor for AD [196]. Cognitive ability can decline faster in AD patients with high levels of cholesterol [197]. Moreover, a high-cholesterol diet has been shown to induce disruption of the BBB by reducing the expression of tight junction proteins [198]. High cholesterol levels can also promote the binding of APPs to lipid rafts and be decomposed into Aβ through the amyloidogenic pathway [199]. Therefore, the levels of cholesterol can lead to the generation of Aβ. These studies suggest that cholesterol can have different mechanisms contributing to the overall pathogenesis of AD.

### 3.7. Hypoxia

Hypoxia is also associated with the development of dementias like AD [200]. Hypoxia leads to the formation of Aβ by modulating APP metabolism. Studies show that hypoxia induces the expression of ΒACE1, and this promotes the production of Aβ [201]. Particularly, the promoter of BACE1 contains a hypoxic response element where HIF1α can bind during hypoxia. Therefore, HIF1α is postulated to be crucial for the induction of BACE1 and the formation of Aβ [201]. Studies have also shown that hypoxia decreases Aβ-degrading enzymes, affecting the clearance of Aβ [202,203].

Approximately 30% of AD cases can be attributable to vascular pathologies like infarct, arteriosclerosis, and amyloid angiopathy [1,3]. Evidence from epidemiologic, neuroimaging, and neuropathological studies show that vascular risk factors are associated with an increased risk of AD [204,205]. Several observational studies showed that elevated blood pressure in middle age was linked to an increased risk of AD [206,207]. However, studies with a longer follow-up period show that low blood pressure in late life can be associated to the development of AD [208]. There is also a 90% or higher co-incidence of cerebral amyloid angiopathy and AD. Stroke and brain infarcts are also associated with an increased risk of dementia and AD [204]. Studies suggest that cerebrovascular lesions and neurodegenerative changes in the brain coexist and may promote the clinical expression of dementia [209].

### 3.8. Mitochondrial Dysfunction

Neurons are high-energy requiring cells that depend on mitochondria for various functions, including generating action potentials, neural transmissions, and axonal transport [210]. Mitochondria provide more than 90% of the total ATP produced [211]. Studies done on induced pluripotent stem cells have shown that these cells shift from glycolysis to oxidative phosphorylation (OXPHOS) when differentiating into neurons, suggesting the importance of mitochondria for neuronal development [212]. Mitochondria are known to be important in axogenesis and neuronal polarity. Depletion of mitochondrial DNA has been shown to prevent axon formation [213]. One study proposed that mitochondria help increase the recovery of synaptic transmissions during high synaptic activity by sequestering Ca^2+^ [214,215]. Data from a genome-wide transcriptomic study and Western blot analysis showed that nuclear genes influencing mitochondrial energy metabolism are under-expressed in AD [22]. Overall, mitochondria play a crucial role in powering various functions within neuronal cells.

However, ample evidence shows that mitochondrial dysfunction plays a key role in developing AD [210]. For example, cells treated with Aβ induce mitochondrial-targeted Aβ accumulation, leading to cellular death [216]. Confocal microscopy has also shown colocalization of Aβ with complex II of the ETC [210]. Studies have demonstrated that the APP accumulates in the mitochondrial import channels, causing an increase in H_2_O_2_ [217]. Aβ intracellular accumulation occurs prior to Aβ extracellular deposition implying its early role in the development of AD [218]. Some studies have also shown that inhibiting mitochondrial function pushes APP processing to Aβ production [219,220].

Apart from the direct impact of Aβ in mitochondria, studies have also shown that mitochondrial DNA is defective in elderly and AD patients [221]. One commonly known theory of AD progression involves the mitochondrial cascade hypothesis, which proposes that a person’s genes determine their baseline mitochondrial function. Various other factors can then influence the rate at which mitochondrial function changes, contributing to AD progression [222]. Early AD specimens have shown a down-regulation of mitochondrial genes in complex I of the electron transport chain [223]. Furthermore, oxidative damage is associated with damaged mtDNA [224].

### 3.9. Oxidative Stress

The ETC consists of complexes I, II, III, IV, and V, which work on catalyzing the phosphorylation of adenosine diphosphate (ADP) to adenosine triphosphate (ATP) [225]. To generate ATP, complex I and II of the ETC must first oxidize NADH and FADH_2,_ respectively [226]. Electrons are then transferred to ubiquinone (coenzyme Q) and from there to complex III. From complex III, electrons are further transferred to cytochrome c and complex IV, where O_2_ is reduced into H_2_0. Finally, ATP is produced by the proton gradient produced from complexes I, III, and IV via complex V. This reduction of O_2_ sometimes leads to a small amount of superoxides [225,226]. These superoxides make up some of the potent oxidants that are called reactive oxygen species (ROS).

Oxidative stress is an imbalance of pro and antioxidants, leading to an increase in reactive nitrogen species (RNS) and ROS [227]. Mitochondria are the primary source of toxic free radicals, which are a product of normal cellular respiration. In normal conditions, about 1–5% of oxygen is converted to ROS [225]. The major sources of mitochondrial ROS production can be attributed to two factors: the first is high NADH/NAD ratio in the matrix and the second is highly reduced coenzyme Q along with high proton gradient and no ATP synthesis [228]. Importantly, various enzymes can quench ROS, but if the amount of free radicals exceeds the neuronal capacity, then oxidative stress, mitochondrial damage, and neuronal damage can occur [229]. MtDNA is one known target of damage from oxidative stress that can continue to exert its effect by downregulating specific mitochondrial proteins. Furthermore, protein oxidation and nitration are also modifications produced in response to oxidative stress. These alterations can affect metabolic enzymes within the ETC [225]. In neurons, these alterations in enzymes might affect their function and lead to neurodegeneration. In AD, oxidative damage is associated with the accumulation of Aβ and NFTs [108].

Well-studied targets of oxidative stress are lipids. Studies have shown that cells treated with Aβ increase lipid peroxidation [230,231]. Lipid peroxidation is initiated by radicals extracting hydrogen from an unsaturated carbon resulting in a carbon centered lipid radical. The lipid radical reacts with O_2_ to form a peroxyl radical (LOO). This peroxyl radical can react with nearby lipids causing a chain reaction of lipid peroxidation [232]. Lipid peroxidation can lead to 4-hydroxynonenal and other oxidation products that can be neurotoxic [230,233].

Apart from lipid oxidation, protein oxidation has been studied in AD. Oxidized proteins can lead to conformational changes, resulting in loss of structural and functional activity [234]. Particularly, Aβ can increase protein oxidation. A proteomic study revealed that 14-3-3ζ and glyceraldehyde-3-phosphate dehydrogenase are oxidized in neurons treated with Aβ [235]. The oxidation of these proteins can lead to some of the commonly known alterations of AD, such as NFTs and glucose hypometabolism.

### 3.10. Glucose Hypometabolism

The brain consumes about 25% of the total body glucose in the resting awake state [236]. Carbohydrates are a predominant substrate for oxidative metabolism in the brain [237]. Particularly, glucose is considered a dominant energy substrate for the brain [236]. Glucose transportation and intracellular oxidative catabolism contribute to the overall cerebral glucose metabolism [236]. Glucose transportation depends on the BBB and the glucose transporters. Astrocytes are known to take up glucose from the blood generate lactate for neuronal energetics [238]. Neurons also have different glucose transporters (GLUTs) that help uptake glucose from the blood [236,239]. The oxidative catabolism depends on glycolysis, pentose phosphate pathway, Krebs cycle, and oxidative phosphorylation [240]. Alterations in these processes might affect the overall metabolism of glucose in the brain.

A known feature of Alzheimer’s disease is the reduction of the cerebral metabolic rate of glucose. FDG-PET studies have shown decreased glucose metabolism, which correlates with AD’s severity [241]. The reduction in the cerebral metabolic rate of glucose is also present in pre-symptomatic individuals that carry the autosomal dominant mutations of familial AD [242,243]. Lee et al. [244] also identified genes that were dysregulated in both AD and diabetes mellitus, suggesting a common pathophysiology. The cerebral cortex of AD patients have decreased GLUT1 and GLUT3 levels, potentially resulting in decreased glucose transport and glucose hypometabolism [245]. Reduced levels of glucose can contribute to a decline in mitochondrial ATP [236].

### 3.11. Dysregulated Homeostasis of Metals and Heme

Evidence also suggests that iron (Fe), copper (Cu), and zinc (Zn) play a role in AD by increasing oxidative stress [227]. The BBB tightly regulates the concentration of Cu, Zn, and Fe. However, increased amounts of Cu, Zn, and Fe have been reported in AD brains. Although there is some debate on whether Fe and Cu are significantly upregulated, several studies have mentioned the dysregulated homeostasis of metals in AD [246,247]. Studies have shown that Aβ plaques contain Cu, Fe, and Zn [247,248]. Furthermore, Aβ can reduce Fe(III) or Cu(II) to produce H_2_O_2_, contributing to oxidative stress in AD [249]. Some studies have even suggested that these trace metals can promote Aβ aggregation. SH-SY5Y cells treated with Fe3+ caused accumulation of APP and B-secretase, leading to increased Aβ42 [250,251].

Another iron containing molecule that has been associated with AD is heme. Heme, also known as iron-protoporphyrin IX, is an essential nutrient involved in various physiological and disease processes [252]. A study by Faux et al. [253] found that people with AD had lower hemoglobin levels, mean cell hemoglobin concentration, and packed cell volume relative to healthy controls. Participants in this study showed a strong association between anemia and AD, suggesting that hemoglobin production might be defective in AD patients [253]. Similarly, in 2013 a study analyzing 2552 older adults found that anemia is associated with an increased risk of developing dementia [254].

**Figure 3:**
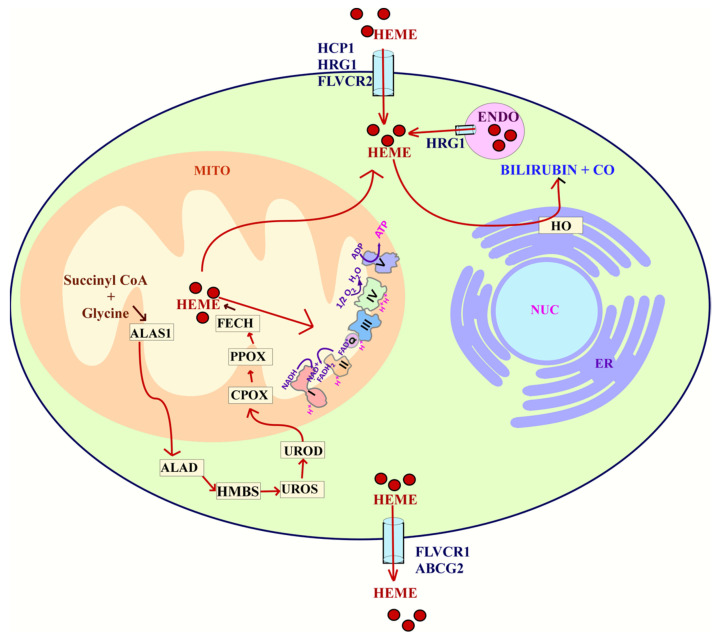
Heme flux. The uptake of heme is mediated by HCP1, HRG1, and FLVCR2. Heme can also be synthesized through an 8 enzyme step reaction that is carried out both in the cytoplasm and mitochondria. Heme acquired either by uptake or synthesis can be utilized for complexes in the ETC. Heme can also be degraded into bilirubin and carbon monoxide (CO) in a process that involves HO. Finally, the export of heme is carried out by FLVCR1 or ABCG2. Abbreviations: heme carrier protein 1 (HCP1), ferrochelatase (FECH), coproporphyrinogen-III oxidase (CPOX), uroporphyrinogen III decarboxylase (UROD), uroporphyrinogen III synthase (UROS), hydroxymethylbilane synthase (HMBS), delta-aminolevulinic acid dehydratase (ALAD), Delta-aminolevulinate synthase 1 (ALAS1), feline leukemia virus subgroup C receptor-related protein 1 (FLVCR1), ATP-binding cassette super-family G member 2 (ABCG2), nucleus (NUC), endoplasmic reticulum (ER), endosome (ENDO), Feline leukemia virus subgroup C cellular receptor family, member 2 (FLVCR2), heme oxygenase (HO), mitochondria (MITO).

Heme in cells is acquired through two main processes: uptake or synthesis (Figure 3). Heme is synthesized in an eight-step process that involves both the mitochondria and cytosol. In the first step, succinyl-CoA and glycine are utilized in the mitochondria to make δ-aminolevulinic acid (ALA) [255]. This step is initiated by the rate limiting enzyme ALAS1. Once ALA is made, it is exported to the cytosol. Then, ALA dehydratase (ALAD) catalyzes the condensation of ALA to form porphobilinogen (PBG). Porphobilinogen deaminase (PBGD) then condenses four molecules of PBG to form hydroxymethylbilane (HMB). Uroporphyrinogen III synthase (UROS) then rearranges HMB to form uroporphyrinogen III. This is converted to coproporphyrinogen III by uroporphyrinogen decarboxylation (UROD). Coproporphyrinogen III can then go into the mitochondria for the next steps of heme synthesis. Through decarboxylation and oxidation, coproporphyrinogen oxidase (CPOX) forms protoporphyrinogen IX [256,257]. Finally, in the last step, ferrochelatase (FECH) inserts iron into protoporphyrin IX to form heme [256].

The uptake and homeostasis of heme involve several transporters such as HCP1, HRG1, FLVCR1, FLVCR2, and ABCG2. The import of intracellular heme is mediated by the heme carrier protein 1 (HCP1), Feline Leukemia Virus subgroup C 2 (FLVCR2), and the heme-responsive gene 1 (HRG-1) [258]. The export of heme is regulated by the ATP binding cassette subfamily G member 2 (ABCG2) and the Feline Leukemia Virus Subgroup C Receptor (FLVCR1) [259]. These transporters help maintain intracellular levels of heme.

Heme is known to be involved in neuronal development. Zebrafish deficient in HRG-1 have shown impaired neuronal growth and differentiation [258,260]. Inhibition of FLVCR2 can cause a lack of complexes III and IV of the ETC [258,261,262]. Furthermore, studies done on PC12 cells showed that inhibiting heme synthesis significantly impairs neuronal development [263]. Heme deficiency can also cause a decrease in phosphorylation, expression, and function of the NMDA receptor in neurons [264]. Furthermore, complexes II, III, and IV of the ETC require heme in order to function [265]. Considering the importance of mitochondria in neurons, heme plays a major role in neuronal function. Consequently, impaired heme metabolism might play a crucial role in AD.

**Figure 4:**
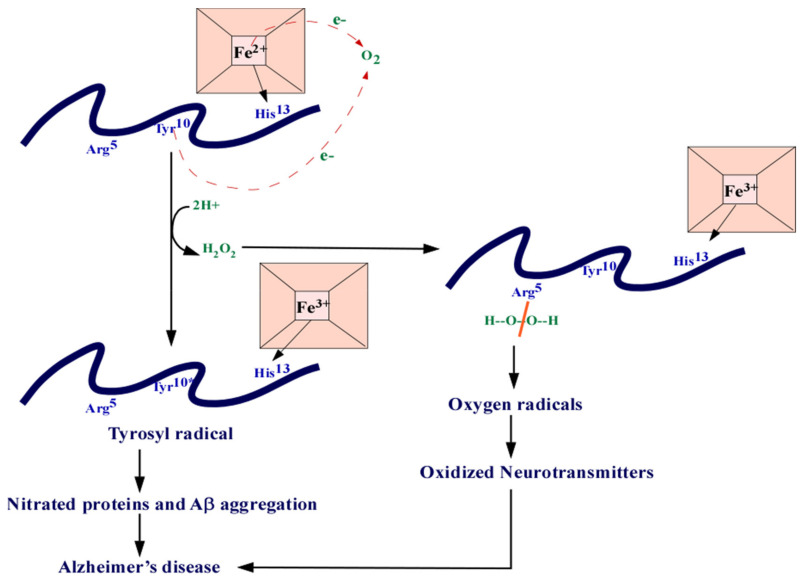
The heme-Aβ complex. Heme can bind to Aβ at the His13 residue. The residues can donate one electron each which reduces O2 into H202. The Arg5 residues can split the H_2_O_2_ and generate oxygen radicals that are responsible in the nitration of proteins. The ROS can also cause oxidized neurotransmitters. Abbreviations: amyloid beta (Aβ), arginine (Arg), tyrosine (Tyr), histidine (Hys), iron (Fe), electron (e-).

Perturbations in heme metabolism can affect the ETC causing loss of complex IV, dimerization of APP, oxidative stress, and cell death [266]. A study by Sankar et al. [267] also found that heme can suppress the Aβ42-mediated inflammatory activation of astrocytes, decreasing Aβ clearance. These are all characteristic alterations seen in AD. Heme can also bind to Aβ, forming a complex that prevents Aβ aggregation (Figure 4). This complex is known to have peroxidase activity that oxidizes neurotransmitters, serotonin, and DOPA, providing a link between heme and the oxidative stress seen in AD [30]. The binding of heme to Aβ might also lead to a deficiency in heme required for various cellular functions. For example, inducing heme deficiency in cells can result in APP dimers and loss of complex IV of the ETC [31,268]. The decrease in complex IV can also cause oxidative stress [268]. Studies have suggested that the iron accumulation seen in AD could be a result of heme deficiency [31]. Furthermore, studies have also shown that ALAS1 is significantly reduced in AD brain [264].

Heme degradation has also been shown to be affected in studies of AD. Heme degradation requires the enzyme Heme oxygenase (HO) to produce biliverdin, carbon monoxide, and iron. The biliverdin produced from heme degradation can then be reduced by biliverdin reductase (BVR) to form the powerful antioxidant bilirubin [269]. There are three known isoforms of HO: HO-1, HO-2, and HO-3. HO-1 is an inducible form of HO induced by various factors, including hypoxia. HO-2 is a constitutive isoform highly expressed in the brain. HO-3 does not have enzymatic activity [269]. The role of HO in AD has been debated, but various studies have implicated its association with AD. For example, cells containing the *APOE4* allele can increase the anti-inflammatory protein HO-1 [270]. Upregulation of HO-1 in AD can lead to the accumulation of iron seen in AD [271]. However, studies have also proposed the protective role of HO-1 in reducing ROS by producing bilirubin [272]. Furthermore, some studies have attributed the oxidative stress seen in AD to the downregulation of BVR-A, the enzyme involved in producing bilirubin [273]. APP can also interact with HO inhibiting its activity and increasing neurotoxicity [274]. Recent studies have also shown that ALAS1 and HO-2 are selectively decreased in AD patients and mice. These studies also showed that Aβ reduces the levels of HO-2 and heme degradation [29]. Regardless of these results, more studies should be done to analyze the temporal changes of heme flux and how they contribute to the progression of AD.

## 4. Models of AD

Mouse models are one of the most important tools for analyzing a wide array of diseases. They provide insight into the mechanisms underlying different diseases and can help develop treatments. Similarly, cellular models have also been useful in examining disease progression. Various models of AD have been developed to understand and characterize the molecular changes that occur in AD. Most importantly, these models can help elucidate the early and causative factors that are crucial in the development of AD.

### 4.1. Cell Models

Various cells and cell lines have been established to emulate the phenotypic and molecular characteristics of neurons. Primary cell cultures are used as a model for neuronal cells. However, these cells are not homogenous nor immortal. Therefore, working with these cells is more complicated. Culturing primary neurons requires the separation of the different cell types [275]. These cells also need to be generated from embryonic or early postnatal brains [276]. The PC12 cell line has also widely been used as a model for neuronal differentiation. The PC12 cell line was initially isolated from a tumor in the adrenal medulla of a rat [277]. These cells can differentiate into sympathetic ganglion neurons when cultured with nerve growth factor (NGF) [278]. The SH-SY5Y cell line is a neuroblastoma cell line commonly used to model neurons because it can be differentiated into neuronal cells. This cell line was generated from the parental neuroblastoma cell line SK-N-SH and was derived from a bone marrow biopsy with both neuroblast-like and epithelial-like cells [279]. These cells are human-derived and therefore express human proteins that are not expressed in rodent primary neurons. The SH-SY5Y cells can be differentiated using different mechanisms but usually contain retinoic acid (RA) and specific neuronal growth factors such as brain-derived neurotrophic factors (BDNF) and NGF [280,281,282,283,284]. Differentiation of these cells produces extension of neuritic processes, increased electrical excitability, and induction of various neuron-specific proteins and enzymes, making them a suitable model for neurons [284]. For these neuronal cells to serve as a model for AD, they are usually treated with Aβ [285,286]. Other researchers have utilized these cell lines and transfected them with mutated or wild type forms of APP [287]. This helps visualize the effect of Aβ on neurons.

More recently, studies have utilized stem cells as an alternative for culturing primary neurons. Induced expression of specific genes can reprogram patient-derived somatic cells into pluripotent stem cells. From this, neural progenitor cells are generated [288]. These cells can be further differentiated into mature neurons with the addition of various growth factors [288]. These stem cells can produce electrophysical characteristics and provide an alternate strategy to create functional neuronal networks [289]. Furthermore, because these cells are patient-derived, various AD-related mutations can be analyzed. For example, induced pluripotent stem cells (iPSC) expressing the *APOE4* allele or *PSEN1* mutations can provide a good model for analyzing AD. One study carried out in 2013 generated FAD and SAD iPSC lines and differentiated these cells into neural cells [290]. This model was useful in understanding whether these oligomers could cause cellular stress and lead to AD pathogenesis [290]. IPSC lines generated from *APOE3/3* and *APOE4/4* subjects have also been useful in illuminating the role of *apoE4* in neurons [291]. The conversion of *APOE4/4* to *APOE3/3* lead to a decrease in the level of *APOE* fragmentation and Aβ40 and Aβ42 secretion into the culture medium [291]. *APOE4/4* neurons also generated increased levels of phosphorylated tau and GABAergic neuron degeneration [291]. Other iPSC lines have been generated to characterize the pathogenesis of sporadic AD, including those mentioned in two 2019 studies carried out by Diaz-Guerra et al. [292]. Despite the importance of iPSC lines for understanding the molecular mechanisms of AD, there are various limitations in using this model. For example, the reprogramming of iPSC lines can cause de novo mutations [293]. This model also has an uncontrolled genetic background and has limited cell–cell interactions [293].

### 4.2. Mouse Models

Although many cases of AD are sporadic (SAD), there are FAD mutations that can mimic the clinical and pathological characteristics of SAD. The familial cases offer a genetic lesion that can be used to model AD in transgenic mice. For example, the first approach to generating these transgenic mice utilized a platelet-derived growth factor-β promoter to drive a human *APP* that contained the V717F mutation. This line had an elevated production of APP protein and Aβ [294]. Other transgenic lines have been developed utilizing similar approaches of incorporating strong promoters to drive APP expression. PS1 FAD mutant transgenic lines have also been generated utilizing similar promoters. However, these lines need to be crossed with APP lines to form a more extensive production of Aβ [294]. The APPPS1 mouse model, for example, contains both the *APP* KM670/671NL mutation and the *PSEN1* L166P mutation, both under the neuron-specific Thy1 promoter [295]. These mice start showing cerebral amyloidosis at 6–8 weeks and contain a high Aβ42 to Aβ40 ratio [295].

The 5xFAD line is another commonly used model of AD that expresses the human *APP* and *PSEN1* transgenes with five AD mutations. This line expresses the Swedish (K670N/M671L), Florida (I716V), and London (V717I) mutations in *APP*, and the M146L and L286V mutations in *PSEN1* [11]. They start to accumulate intraneuronal Aβ42 as young as 1.5 months of age and have an age-dependent decrease in synaptic activity [11]. These are some of the most commonly used mouse models for AD, but several others have been developed to analyze the different pathologies of AD [294,296]. Utilizing these mouse models can be useful because they provide a controlled genetic background. However, they can generate artifacts and unwanted genetic alterations that might affect the overall interpretation of the results [293].

Another model used for studying AD pathogenesis is the xenograft mouse model, in which human iPSC-derived cells are transplanted into the mouse brain [293]. This provides a 3D matrix for human cells and helps reproduce many human features. In a 2017 study, cortical precursor cells were implanted into newborn mice to understand whether Aβ generated in an AD mouse model can induce full AD pathology in non-manipulated human neurons [297]. This xenograft model showed that transplanted neurons show remarkable signs of neurodegeneration not detected in the mouse host brain [297]. This suggests that human neurons respond to Aβ pathology differently than mouse cells. Although these xenograft models can be useful for studying AD, they do have several limitations. For example, the human-to-mouse cell interactions might affect the overall results of the experiments. Furthermore, this model requires immune-compromised mice, which can affect the outcome and interpretation of the findings [293].

## 5. Conclusion

**Figure 5:**
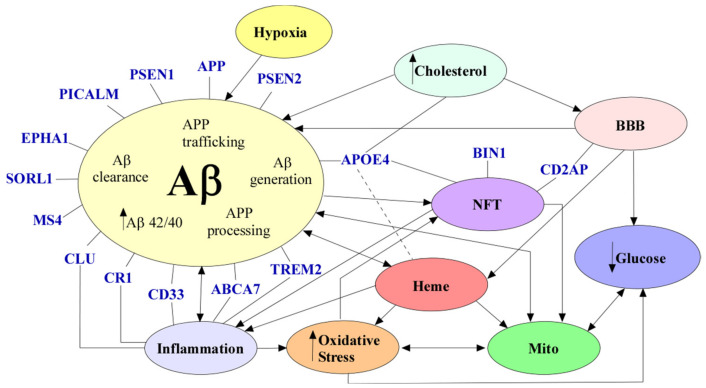
The mechanisms of AD. Aβ (in beige) can cause inflammation (in blue), NFTs (in purple), dysregulated heme metabolism (in red), and mitochondrial dysfunction (in green). Increased cholesterol (in light blue) levels have been seen to cause BBB (in pink) disruption and Aβ accumulation. A dysfunctional BBB can cause decreased Aβ clearance, increased heme, and decreased glucose (in dark blue). NFTs have been seen to cause mitochondrial dysfunction and inflammation (in blue). Oxidative stress (in orange) can cause mitochondrial dysfunction, decreased glucose, and NFTs. Glucose hypometabolism can cause decreased ATP. Inflammation can cause decreased amyloid beta clearance and oxidative stress. Mitochondrial dysfunction can cause oxidative stress, Aβ generation and glucose hypometabolism. Hypoxia (in yellow) can cause Aβ generation. Dysregulated heme homeostasis can cause oxidative stress, mitochondrial dysfunction, and dimerization of APP. All genetic risk factors are denoted in blue. Variants *in PICALM, PSEN1, APP, PSEN2, EPHA1, SORL1, MS4, TREM2, CLU, CR1, CD33, ABCA7*, and *APOE4* can all indirectly or directly cause Aβ accumulation. *CLU, CR1, CD33, ABCA7*, and *TREM2* have a role in inflammation. *APOE, BIN1* and *CD2AP* variants can cause NFTs. Abbreviations: amyloid beta (Aβ), neurofibrillary tangles (NFT), blood–brain barrier (BBB), mitochondrial dysfunction (mito).

AD is a neurological disease that affects millions of people throughout the world, and despite countless studies, there are no effective treatments for this disease. Aβ accumulation, NFTs, neuronal loss, dysfunctional BBB, inflammation, defective cholesterol metabolism, hypoxia, mitochondrial dysfunction, oxidative stress, glucose hypometabolism, and dysregulated heme homeostasis are alterations commonly seen in AD (Figure 5). However, there is no consensus on which factor is instrumental in causing AD. One specific factor that seems to link most of the alterations seen in AD is the dysregulation of heme homeostasis. As previously described, heme is imperative for neuronal function, and dysfunctional heme metabolism can cause mitochondrial dysfunction, oxidative stress, and even the accumulation of Aβ seen in AD. Nevertheless, more studies should be conducted to understand the role of heme and heme metabolism in AD pathology.

The discovery of the genetic risk factors associated with AD has allowed researchers to design specific models of AD that can help glean the molecular changes that occur in AD patients. The cell and mouse models previously described can serve as a suitable platform to analyze the presumptive causative factors of AD. The neuronal cell lines and stem cells can also provide an insight into the different metabolic pathways essential for neuronal function. Therefore, utilizing both of these models can be instrumental in understanding AD pathology.

## Data Availability

No new data were created or analyzed in this study. Data sharing is not applicable to this article.
